# Granule Cells Constitute One of the Major Neuronal Subtypes in the Molecular Layer of the Posterior Cerebellum

**DOI:** 10.1523/ENEURO.0289-21.2022

**Published:** 2022-05-31

**Authors:** Moushumi R. Dey, Kirthan Reddy, Hiroichi Yoshida, Naoko Nishiyama, Boris V. Zemelman, Hiroshi Nishiyama

**Affiliations:** Center for Learning and Memory, Department of Neuroscience, The University of Texas at Austin, Austin, TX 78712

**Keywords:** cerebellum, development, ectopic neurons, granule cells, migration

## Abstract

The migration of neurons from their birthplace to their correct destination is one of the most crucial steps in brain development. Incomplete or incorrect migration yields ectopic neurons, which cause neurologic deficits or are negligible at best. However, the granule cells (GCs) in the cerebellar cortex may challenge this traditional view of ectopic neurons. When animals are born, GCs proliferate near the pia mater and then migrate down to the GC layer located deep in the cerebellar cortex. However, some GC-like cells stay in the molecular layer, a layer between the pia mater and GC layer, even in normal adult animals. These cells were named ectopic GCs nearly 50 years ago, but their abundance and functional properties remain unclear. Here, we have examined GCs in the molecular layer (mGCs) with a specific marker for mature GCs and transgenic mice in which GCs are sparsely labeled with a fluorescent protein. Contrary to the previous assumption that mGCs are a minor neuronal population, we have found that mGCs are as prevalent as stellate or basket cells in the posterior cerebellum. They are produced during a similar period as regular GCs (rGCs), and *in vivo* time-lapse imaging has revealed that mGCs are stably present in the molecular layer. Whole-cell patch-clamp recordings have shown that mGCs discharge action potentials similarly to rGCs. Since axonal inputs differ between the molecular layer and GC layer, mGCs might be incorporated in different micro-circuits from rGCs and have a unique functional role in the cerebellum.

## Significance Statement

During brain development, neurons migrate away from the place they are born to their correct destination. A defect in this process yields ectopic neurons, i.e., abnormally positioned neurons, which have been considered harmful and studied mostly in the context of brain diseases. Here, we show that abundant granule cells (GCs) in the cerebellum are located ectopically in normal, healthy animals. These neurons are functionally mature and discharge action potentials similarly to conventional GCs. The seemingly ectopic GCs in the normal cerebellum may not be harmful or negligible. Rather, their abundance and distinct location suggest that they might play a unique functional role in the cerebellum via previously unconsidered neuronal connections.

## Introduction

Neuronal migration is an elaborate developmental program, and a failure in this process often causes severe motor and cognitive dysfunction because of brain malformation. Therefore, ectopic neurons, abnormally positioned neurons, have been studied mostly in their relation to neurologic diseases (Takao et al., 2001; Pierce et al., 2005; McCloskey et al., 2006; Watrin et al., 2015). However, some neurons are placed ectopically even in the normal, healthy brain. Well-known examples are granule cells (GCs) in the hippocampal dentate gyrus (DG) and cerebellar cortex (Chan-Palay, 1972; Palay and Chan-Palay, 1974; Gaarskjaer and Laurberg, 1983; Scharfman et al., 2007). These seemingly harmless ectopic neurons have not been paid much attention, and it remains largely unclear whether they are just tolerable errors or play some roles in brain function.

The mature cerebellar cortex consists of four distinct layers: the molecular layer, Purkinje cell layer, GC layer, and white matter in this order from the surface (Palay and Chan-Palay, 1974; Altman and Bayer, 1996). GCs migrate down from the surface to the GC layer during development, but some GCs have been found in the molecular layer of the normal adult cerebellum in various animal species (Brzustowicz and Kernohan, 1952; Spacek et al., 1973; Palay and Chan-Palay, 1974; Lafarga and Berciano, 1985; Berciano and Lafarga, 1988).

Thus far, those molecular layers GCs (mGCs) have been studied using nonspecific histochemical techniques, such as hematoxylin-eosin staining and Nissl staining. With these staining techniques, mGCs can be unambiguously identified only when they form a clear, distinguishable cluster near the pia mater. If individual mGCs are dispersed throughout the molecular layer, they are difficult to identify because their staining pattern appears similar to that of molecular layer interneurons (MLIs), i.e., stellate cells and basket cells. As a consequence, the number of mGCs has likely been underestimated. Indeed, Golgi staining revealed GCs distributed throughout the molecular layer (Lafarga and Berciano, 1985; Berciano and Lafarga, 1988). Although Golgi staining is nonspecific, it highlights the entire structure of sparsely labeled cells, allowing cell-type identification. The dendritic structure of mGCs is slightly different from regular GCs in the GC layer (rGCs); hence they were considered immature forms of GCs (Lafarga and Berciano, 1985). Combined with the assumption that GCs are a minor population in the molecular layer, mGCs have been regarded as a harmless error in migration (Berciano and Lafarga, 1988).

It may be too early to draw such a conclusion because previous studies of mGCs were performed several decades ago before a GC-specific marker was established. Therefore, the actual abundance of mGCs remains unclear. Furthermore, the functional properties of mGCs have not yet been studied because identifying them in living tissues was nearly impossible. Now, the GABA_A_ receptor α6 subunit (GABA_A_Rα6) is an established marker for mature GCs (Kato, 1990; Laurie et al., 1992b; Nusser et al., 1996; Mellor et al., 1998). A transgenic mouse line in which GCs are selectively labeled with fluorescent proteins is available for functional characterization of mGCs (Huang et al., 2013; Shima et al., 2016; Dhar et al., 2018). Using these new tools, we have found that mGCs, GABA_A_Rα6-positive cells, are as prevalent as stellate or basket cells in the molecular layer of the posterior cerebellum. Whole-cell patch-clamp recordings and *in vivo* time-lapse imaging suggest that mGCs are stable components in the molecular layer, potentially participating in local synaptic circuitry in a different way from rGCs.

## Materials and Methods

### Animals

C57BL/6J (B6) mice and TCGO transgenic mice (males and females) were used for this study. TCGO is one of >200 enhancer trap lines that label small subsets of neurons (Shima et al., 2016). A fraction of cerebellar GCs expresses mCitrine fluorescent protein in TCGO mice (Huang et al., 2013; Dhar et al., 2018), although it is unknown why mCitrine expression is GC-specific and why the labeling is sparse. TCGO line was backcrossed to B6, and the experimental animals were obtained from TCGO × B6 and TCGO × TCGO. The mice were four-month-old or older unless otherwise stated. All animal procedures were performed in accordance with the University of Texas at Austin, Institutional Aimal Care and Use Committee’s regulations.

### 5-Bromo-2'-deoxyuridine (BrdU) cell proliferation assay

BrdU was subcutaneously injected into B6 pups (150 mg/kg). Individual pups received two pulses of injection (4-h interval) either at postnatal day (P)4, P8, or P12 and were returned to the home cage with their mother. They were sacrificed approximately two months after the injection and processed for immunohistochemistry as described below but with an additional antigen retrieval step. For antigen retrieval, cerebellar sections were first incubated with 2N HCl at room temperature for 30 min. The HCl was washed thoroughly with PBS (in mm; 137 NaCl, 2.7 KCl, 8 Na_2_HPO_4_, and 2 KH_2_PO_4_, pH 7.4) containing 0.5% Triton X-100 (PBST) before proceeding to the blocking step of immunohistochemistry. Image acquisition and analysis were performed as described below.

### Immunohistochemistry

B6 or TCGO mice were anesthetized with an intraperitoneal injection of ketamine/xylazine (100/10 mg/kg) and then intracardially perfused with 4% paraformaldehyde in PBS. The brains were extracted postperfusion and immersed in the same fixative overnight at 4°C. After washing the tissues with PBS, sagittal sections of the cerebellum (30–60 μm in thickness) were cut using Microm HM650V vibration microtome (Thermo Fisher Scientific). The sections were washed with PBST and blocked for at least 1 h at room temperature in PBST containing 5% normal donkey serum. Following the blocking step, the sections were incubated with primary antibodies overnight at 4°C, then washed with PBST and incubated with secondary antibodies for at least 2 h at room temperature. Sections were then washed with PBST and mounted with mounting media (Fluoromount-G, SouthernBiotech). The list of the primary and secondary antibodies is shown below.

Primary antibodies: anti-GABA_A_Rα6 rabbit polyclonal antibody (1:500, #224603, Synaptic Systems), anti-parvalbumin (PV) mouse monoclonal antibody (1:2000, ab277625, Abcam), anti-BrdU rat monoclonal antibody (1:500, ab6326, Abcam), anti-NG2 rabbit polyclonal antibody (1:500, AB5320, Millipore Sigma). Secondary antibodies: donkey anti-rabbit IgG H&L Alexa Fluor 488 (1:500, ab150073, Abcam), donkey anti-mouse IgG H&L Alexa Fluor 568 (1:500, ab175472, Abcam), donkey anti-rat IgG H&L Alexa Fluor 568 (1:500, ab175475, Abcam), donkey anti-rabbit IgG H&L Alexa Fluor 647 (1:500, ab150075, Abcam), donkey anti-mouse IgG H&L Alexa Fluor 647 (1:500, ab150107, Abcam).

Two-color detection was performed with the following fluorophore combination: GABA_A_Rα6 (Alexa Fluor 488) – PV (Alexa Fluor 568), GABA_A_Rα6 (Alexa Fluor 488) – BrdU (Alexa Fluor 568), mCitrine – GABA_A_Rα6, PV, or NG2 (Alexa Fluor 647).

### Image acquisition and analysis

The immunostained sections were imaged using Olympus FV1000 laser-scanning confocal microscope (Olympus). Z-stack images were acquired with a 40× water immersion objective lens (0.8 NA) at 1- to 1.5-μm step size with the *x-y* resolution of 0.4–0.6 μm/pixel. Alexa Fluor 488 and Alexa Fluor 568 were excited by 488- and 543-nm lasers, respectively. The green (Alexa Fluor 488) and orange (Alexa Fluor 568) fluorescent emissions were separated by a long-pass filter 560 nm and then filtered by bandpass filters 505–525 and 560–660 nm, respectively. mCitrine and Alexa Fluor 647 were excited by 515- and 635-nm lasers, respectively. The yellow (mCitrine) and red (Alexa Fluor 647) fluorescent emissions were separated by a long-pass filter 640 nm and then filtered by bandpass filters 535–565 and 655–755 nm, respectively. The laser intensity and the sensitivity of the photomultiplier tubes were manually optimized in each field of view (FOV) because the signal intensity and background noise are not always the same across samples and even within a sample.

**Figure 1.**
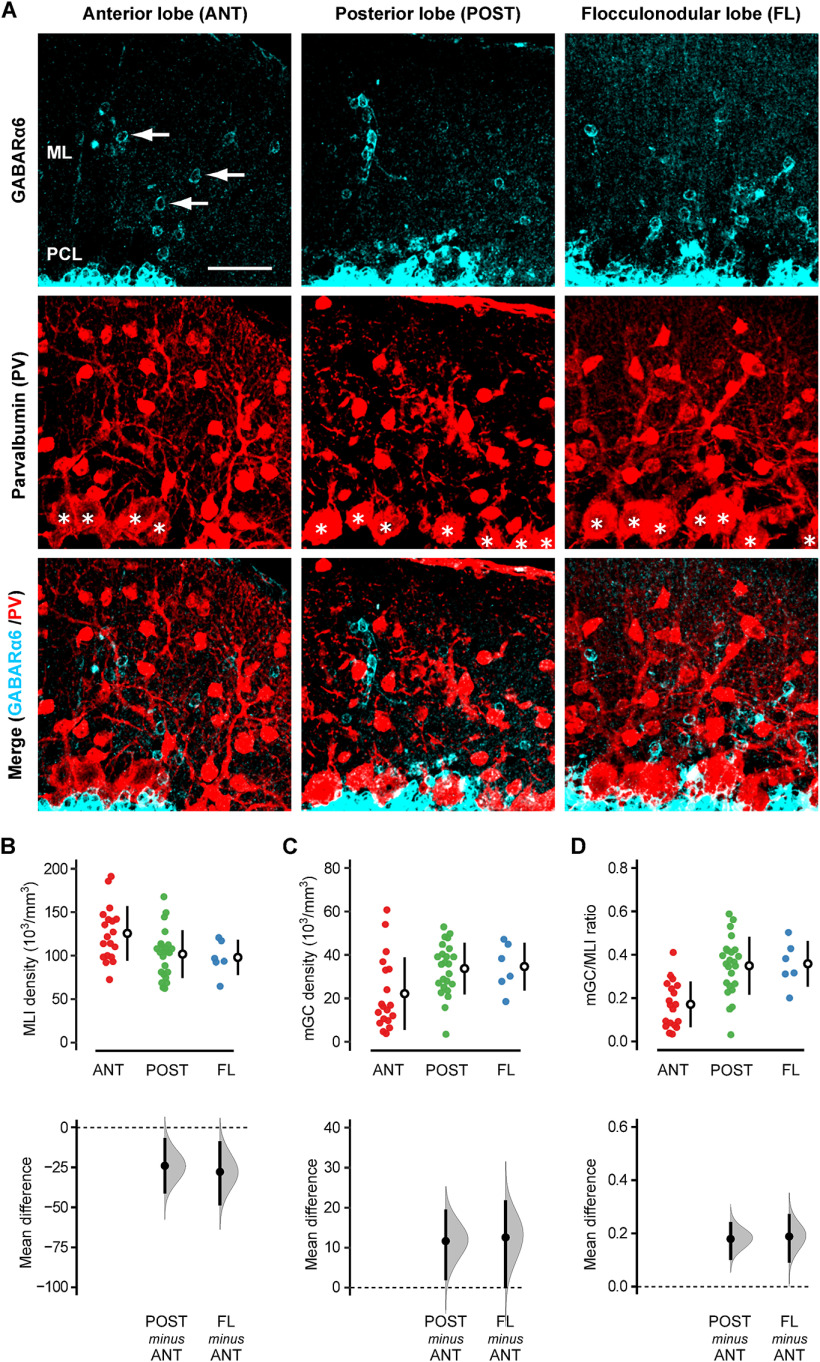
Immunohistochemical detection of mGCs and MLIs. ***A***, The sagittal cerebellar slices of B6 mice were double-stained with antibodies for GABA_A_Rα6 (cyan, top row) and PV (red, middle row). The merged images are shown in the bottom row. Note that GABA_A_Rα6 signals do not colocalize with the somata of PV-positive cells. The left, middle, and right columns show examples taken from the anterior lobe (ANT; Lobules I–V), posterior lobe (POST; Lobules VI–IX), and flocculonodular lobe (FL; lobule X), respectively. The arrows in the top row indicate examples of mGCs. The asterisks in the middle row indicate Purkinje cell somata. ML, molecular layer; PCL, Purkinje cell layer. Scale bar: 40 μm. ***B–D***, The density of MLIs (***B***), mGCs (***C***), and the mGC/MLI ratio (***D***) were quantified in each lobe, and the mean differences between ANT versus POST and ANT versus FL were estimated by bootstrap resampling. In the top panels, the closed circles indicate individual FOVs taken from ANT (red; *n* = 19), POST (green; *n* = 24), and FL (blue; *n* = 6) of four mice. The open circles and vertical lines indicate the mean and standard deviation (SD), respectively. In the bottom panels, the gray curves indicate the resampled distribution of the mean difference between ANT versus POST (POST minus ANT) and ANT versus FL (FL minus ANT). The closed black circles and vertical lines indicate the observed mean difference and 95% confidence interval, respectively. The horizontal dashed line is the line of zero mean difference. The statistical significance (*p* < 0.05) is assessed whether the 95% confidence interval includes this zero-line or not. Furthermore, the sharpness of the resampled distribution (relative to the mean difference) and the proximity of the confidence interval to the zero-line allow us to infer the certainty of the difference. All the estimation graphics used in this study have the same structure.

To quantify the density of mGCs and MLIs (the mixture or basket and stellate cells) in B6 mice, the number of GABA_A_Rα6-positive cells (mGCs) and PV-positive cells (MLIs) were manually counted in 3D, z-stack images. The FOVs were selected from the anterior lobe (Lobules I–V), posterior lobe (Lobules VI–IX), and flocculonodular lobe (Lobule X). Then, in each FOV, the numbers of mGCs and MLIs were divided by the ML volume to obtain the density. The ML volume (range; 5.8 × 10^−4^ mm^3^ to 28.0 × 10^−4^ mm^3^) was calculated as the ML area × the depth of the z-stack in which counted cells were included. The numbers of animals and FOVs in each lobe are shown in Figure 1 legend.

**Figure 2.**
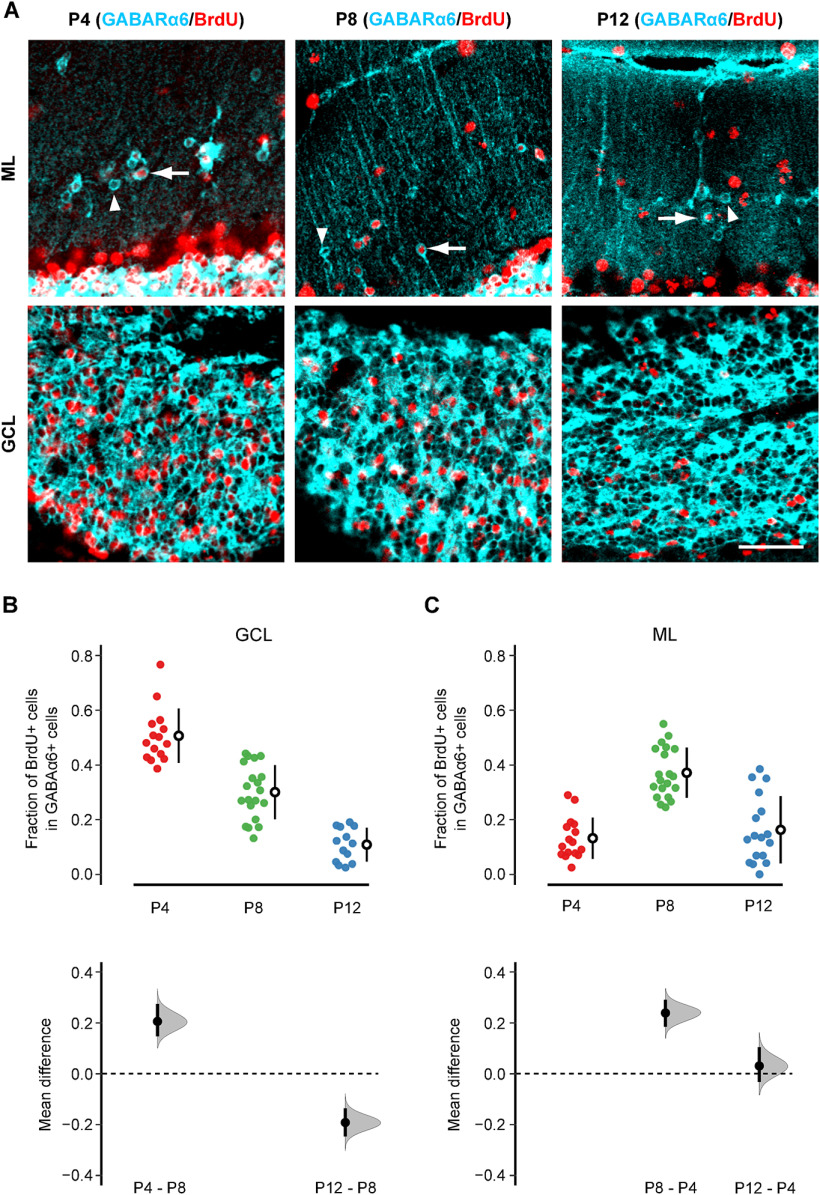
BrdU cell proliferation assay. ***A***, Representative images of BrdU (red) and GABA_A_Rα6 (cyan) double staining in the normal adult cerebellum. The top and bottom rows indicate the images taken from the molecular layer (ML) and GC layer (GCL), respectively. BrdU was injected at P4 (left column), P8 (middle column), or P12 (right column). The arrows indicate examples of BrdU-positive mGCs, whereas the arrowheads indicate examples of BrdU-negative mGCs. Scale bar: 40 μm. ***B***, ***C***, The fraction of BrdU-positive cells in GABA_A_Rα6-positive cells was quantified in the GCL (***B***) and ML (***C***), and the mean differences between the BrdU injection days were estimated by bootstrap resampling. In the top panels, the closed circles indicate individual FOVs obtained from P4 injection (red; *n* = 15 for GCL, 16 for ML from 4 mice), P8 injection (green, *n* = 20 for GCL, 20 for ML from 5 mice), and P12 injection (blue; *n* = 13 for GCL, 17 for ML from 4 mice). The open circles and vertical lines indicate the mean and SD, respectively. In the bottom panels, the estimated mean differences between P8 versus P4 (P4 – P8) and P8 versus P12 (P12 – P8) are shown for the GCL (***B***), and the estimated mean differences between P4 versus P8 (P8 – P4) and P4 versus P12 (P12 – P4) are shown for the ML (***C***). The gray curves indicate the resampled distribution of the mean difference. The closed black circles and vertical lines indicate the observed mean difference and 95% confidence interval, respectively. The horizontal dashed line is the line of zero mean difference.

For BrdU cell proliferation assay in B6 mice, the numbers of GABA_A_Rα6-positive cells and BrdU/GABA_A_Rα6-double-positive cells were manually counted in the molecular layer and GC layer in 3D, z-stack images. Then, the fraction of BrdU/GABA_A_Rα6-double-positive cells in GABA_A_Rα6-positive cells was calculated in each layer. The FOVs were selected randomly from the entire cerebellum. The numbers of animals and FOVs are shown in Figure 2 legend. The average volume for quantification was 14.0 × 10^−4^ mm^3^. The quantification was done by two experimenters, one knew when BrdU was injected, and the other did not.

For the identification of mCitrine-expressing cells, at least four TCGO mice were used for each immunostaining against GABA_A_Rα6, PV, or NG2. The FOVs were selected randomly from the entire cerebellum, and the images were examined whether the immunoreactivity colocalized with mCitrine fluorescence.

### Cranial window preparation

TCGO mice (one month old or older) were anesthetized with 5% isoflurane, and the dose was reduced to 1.5–2% following the induction. A cranial window was created on the dorsal surface of the cerebellar cortex (Lobules VI-VIII) as described in a previous study (Nishiyama et al., 2014). Briefly, the scalp, muscles, and fascia overlying the skull were removed. A small metal bar (12 × 2 mm in length and ∼ 0.9 mm in thickness) was glued to the skull near the lambda with surgical cyanoacrylate (Vetabond, 3M) and dental cement (Ortho-Jet, Lang Dental). The mouse’s head was stabilized by clamping the metal bar to the surgical stage. A dental drill and a small drill bit (0.5-mm tip diameter) were used for etching a rectangular (∼2 × 1.5 mm) craniotomy on the skull. After an opening was made, a coverslip was placed directly on top of the dura and secured in place using the surgical cyanoacrylate and dental cement. The animal was then allowed to recover from the anesthesia and returned to the home cage. Carprofen (5 mg/kg) was administrated for 2 d postsurgery as an analgesic.

### *In vivo* time-lapse imaging

We began long-term two-photon *in vivo* time-lapse imaging one to two weeks after the cranial window preparation. Mice were lightly anesthetized with 1–1.5% isoflurane and securely positioned on a custom-made *x-y* translator under Olympus FV1000 two-photon laser-scanning microscope (Olympus). mCitrine was excited by 920-nm pulsed infrared laser provided by Mai Tai HP DeepSee mode-locked Ti:sapphire laser (Spectra-Physics). The emitted fluorescence was collected with a 25× water immersion objective lens (1.05 NA) and detected by an external gallium arsenide photodetector (GaAsPs; Hamamatsu).

Both mGCs and rGCs express mCitrine in TCGO mice. However, mGCs and rGCs are unambiguously distinguishable because mGCs are found within parallel fiber bundles, which exist only in the molecular layer. Z-stack images (1.5-μm step size, 30–60 planes with the *x-y* resolution of 0.3 μm/pixel) were taken repetitively in the same FOVs once a week until the window clarity diminished or the animals showed a sign of potential health concern. The brain surface vasculature was used to locate the same FOVs roughly, and the position was confirmed and refined by mGC signals, which were stable across time points. We imaged 344 mGCs from 17 mice in total. Among them, 181 mGCs from 10 mice were imaged longer than one month. Since the inability of month-long imaging likely reflects suboptimal surgery, we only focused on those 181 mGCs and tracked their presence in 3D z-stacks across timepoints. Cells located near the top or bottom of a z-stack were excluded from the analysis because those cells might not be captured at every time point because of a slight angular difference in the optical axis.

### Electrophysiology

TCGO mice were deeply anesthetized with isoflurane. The cerebellum was quickly removed after decapitation and submerged in an ice-cold cutting solution containing (in mm): 2.5 KCl, 0.5 CaCl_2_, 7.0 MgCl_2_, 1.25 NaH_2_PO_4_, 25 NaHCO_3_, 205 Sucrose, and 15 glucose bubbled with 95% O_2_ and 5% CO_2_. Parasagittal slices (200 μm thick) were cut using a 7000smz-2 vibration microtome (Campden Instruments), recovered in artificial CSF (ACSF; in mm: 125 NaCl, 2.5 KCl, 1.25, NaH_2_PO_4_, 25 NaHCO_3_, 2 CaCl_2_, 1 MgCl_2_, and 10 glucose) for 30 min at 32°C, and then stored at room temperature until used for recording. Recordings were performed at 32°C.

Whole-cell patch-clamp recordings were made using Multiclamp 700B amplifier (Molecular Devices) and AxoGraph data acquisition software (AxoGraph). The tip of the recording pipettes (7–12 MΩ) was wrapped with Parafilm to reduce the capacitance. Whole-cell current-clamp recordings were performed using the pipettes filled with the intracellular solution containing (in mm): 150 K-gluconate, 10 NaCl, 10 K-HEPES, 3 Na_2_-ATP, 0.3 Na-GTP (305–310 mOsm, pH adjusted to 7.3 with KOH). mCitrine-expressing cells in the molecular layer (mGC+), mCitrine-expressing cells in the GC layer (rGC+), and mCitrine-nonexpressing cells in the GC layer (rGC–) were selected at random, but mGC+ located at the pia mater was avoided. Series resistance was compensated using bridge balance, and pipette capacitance was neutralized. Cells that required >1.5 pF of capacitance neutralization were removed from further analysis. Passive and active membrane properties of the cells were quantified as described below.

### Electrophysiology data analysis

Hyperpolarizing current steps (750 ms, 1–10 pA with 1-pA increment) were injected to quantify resting membrane potentials and input resistance. Resting membrane potentials were obtained as the averaged voltages without holding current. To quantify input resistance, the steady-state voltage deflection by each hyperpolarizing current was obtained as the averaged voltage during the last 200 ms of hyperpolarizing current injection. The voltage deflections were plotted as the function of the amplitude of the injected currents, and input resistance was obtained as the slope of the linear regression line.

Depolarizing current steps (750 ms, 0–35 pA with 5-pA increment) were injected to quantify the excitability of the cells. Action potential threshold was obtained as the voltage where dV/dt exceeds 10 mV/ms. The action potential firing frequency was obtained as the mean firing rate during the first 200-ms period of depolarizing current injection. Input threshold was defined as the minimum current that elicited action potentials. The action potential frequencies were plotted as the function of the amplitude of the injected currents, and the linear regression line was obtained within the domain from the maximum current that did not elicit action potentials and 35 pA. The slope of this line was used as a measure of the input-output relationship.

The measurements were performed with AxoGraph, and the obtained data were processed with custom-written R codes for visualization, linear fit, and statistical analysis.

### Statistical analysis

Bootstrap resampling (5000 resamples) was performed using an R package dabestr (Ho et al., 2019) to present the effect size, i.e., the mean difference between control and test groups, with its resampling distribution and 95% confidence interval. If the 95% confidence interval does not include zero, it is equivalent to *p* < 0.05 in null hypothesis significance testing. This method does not require the sample or population to be normally distributed. Furthermore, showing the effect size with its entire range of distribution allows better inference beyond whether the effect is statistically significant or not (Calin-Jageman and Cumming, 2019; Ho et al., 2019).

Regarding passive and active membrane properties, mGC+ showed a smaller variance than rGC+ and rGC– in the input resistance and current-frequency (input-output) relationship. Bartlett’s test was used in these cases to test the homogeneity of variance, and *p* < 0.05 was considered statistically significant.

## Results

### GC is one of the major neuronal subtypes in the molecular layer of the posterior cerebellum

According to the traditional view of cerebellar anatomy, MLIs (basket cells and stellate cells) are the only neuronal subtypes that have their somata in the molecular layer (Palay and Chan-Palay, 1974; Altman and Bayer, 1996; D’Angelo, 2018). Therefore, we first performed double-immunohistochemistry using the anti-GABA_A_Rα6 antibody and anti-parvalbumin (PV) antibody to quantify how prevalent GCs are, relative to MLIs, in the molecular layer of B6 mice.

GCs express GABA_A_ receptors and the subunit expression pattern changes with developmental stages; GC precursors express the subunits α2, α3, β3, γ1, and γ2, whereas the expression of the subunit α6 (GABA_A_Rα6) is restricted to postmigratory mature GCs (Laurie et al., 1992a). Therefore, GABA_A_Rα6 has been used as a marker for mature GCs (Kato, 1990; Laurie et al., 1992b; Nusser et al., 1996; Mellor et al., 1998).

PV, a slow calcium-binding protein, is expressed in subsets of GABAergic interneurons, and its expression is specific to MLIs, Golgi cells, and Purkinje cells in the cerebellar cortex (Bastianelli, 2003). Among them, Golgi cells are located in the GC layer, and Purkinje cells are located at the bottom of the molecular layer. Within the molecular layer, anti-PV antibody labels Purkinje cell dendrites, but it highlights the soma of MLIs (Fig. 1*A*, middle). Therefore, it has been used to label MLIs in various animal species (Ohshima et al., 1991; Kadowaki et al., 1993; Fortin et al., 1998; Kalinichenko and Pushchin, 2008; Kamiya and Sawada, 2021).

The anti-GABA_A_Rα6 antibody labeled GCs as small, open circles in the molecular layer (Fig. 1*A*, top), which were distinct from PV-positive MLIs (Fig. 1*A*, bottom). We called them mGCs and quantified the density of mGCs, MLIs, and their ratio (mGC/MLI) on parasagittal sections of the cerebellar vermis.

The cerebellum is divided into three lobes along the anterior-posterior axis by two major fissures: the anterior lobe (Lobules I–V), posterior lobe (Lobules VI–IX), and flocculonodular lobe (Lobule X). The density of mGCs and MLIs varied across the FOVs in all three lobes. However, as a population, the anterior lobe had more MLIs (125.7 ± 31.5 × 10^3^/mm^3^, mean ± SD) and fewer mGCs (22.2 ± 16.7 × 10^3^/mm^3^) than the posterior lobe (MLIs: 101.8 ± 27.4 × 10^3^/mm^3^, mGCs: 33.8 ± 11.9 × 10^3^/mm^3^) and flocculonodular lobes (MLIs: 98.0 ± 20.2 × 10^3^/mm^3^, mGCs: 34.7 ± 11.0 × 10^3^/mm^3^). Figure 1*B*,*C* shows these results (top panels) with the mean difference between the anterior lobe and other lobes, with its estimated distribution and 95% confidence interval (bottom panels). Except for the difference in mGC density between the anterior lobe and flocculonodular lobe (Fig. 1*C*, bottom right), these differences were statistically significant, i.e., the 95% confidence interval of the mean difference not including zero. However, even in these statistically significant cases, the 95% confidence intervals contain values close to zero, suggesting that the actual difference could be minimal or uncertain.

The primary purpose of this analysis is to quantify the relative abundance of GCs as compared with MLIs. Therefore, we calculated the mGC/MLI ratio in each FOV (Fig. 1*D*). The ratio was significantly higher in the posterior lobe (0.35 ± 0.13) and the flocculonodular lobe (0.36 ± 0.11) than the anterior lobe (0.17 ± 0.11). Furthermore, the 95% confidence interval was narrow relative to the mean difference, suggesting that the difference is likely real.

It is important to note that MLIs are a mixture of two different neuronal types: basket cells and stellate cells. If the mGC/MLI ratio is 0.35, and the number of basket cells and stellate cells are comparable, the ratio of mGC: basket: stellate is estimated to be 0.35:0.5:0.5 = 0.7:1:1. Therefore, the number of mGCs is estimated to be ∼70% of each MLI subtype in the posterior cerebellum.

### mGCs are produced during a similar period as rGCs

When animals are born, GCs proliferate near the pia mater and form the external GC layer (Rahimi-Balaei et al., 2018). The external GC layer then gradually disappears as postproliferating GCs migrate down through the molecular layer to form the GC layer deep inside the cortex (Altman and Bayer, 1996; Rahimi-Balaei et al., 2018). Although mGCs are speculated to be the last ones to exit the mitotic cycle and thus could not complete their migration to the GC layer (Lafarga and Berciano, 1985), no previous study has tested this hypothesis. Therefore, we performed BrdU cell proliferation assay combined with GABA_A_Rα6 immunostaining to test whether mGCs represent the group of GCs that exit the mitotic cycle last (Fig. 2).

We injected BrdU into B6 pups at P4, P8, or P12. These dates were chosen because P4–P12 covers the almost entire range of GC proliferation as previous studies showed that the fraction of proliferating GCs was nearly 100% at P4, ∼50% at P8, and only 10–20% at P12 in rats (Fujita, 1967). We sacrificed the mice two months after the BrdU injection and double-stained the sections with anti-BrdU antibody and anti-GABA_A_Rα6 antibody (Fig. 2*A*). Then, the fraction of BrdU-positive cells in the population of GABA_A_Rα6-positive cells was quantified in each FOV without distinguishing lobes (Fig. 2*B*, *C*). We did not address potential differences among lobes for the rest of this study.

The fraction of BrdU-positive GCs, i.e., BrdU and GABA_A_Rα6-double-positive cells, showed a steady decline in the GC layer as BrdU was injected at a later time point (Fig. 2*B*). It indicates that our BrdU cell proliferation assay reliably captured the overall time course of GC proliferation. Therefore, this assay can be used to examine the time course of mGC production.

If mGCs are the last group of GCs that exit the mitotic cycle, all of them should be labeled by BrdU regardless of the timing of injection. In this scenario, two possible outcomes are expected. One possibility is that the fraction of BrdU-positive GCs is not affected by the timing of BrdU injection in the molecular layer. However, it is reasonable to assume that the strength of BrdU labeling gradually decreases and eventually becomes undetectable as cells undergo more mitotic cycles after the BrdU injection. Therefore, the other possibility is that the fraction of BrdU-positive GCs increases in the molecular layer as injection timing is delayed toward the end of the GC proliferation phase. As shown in Figure 2*C*, the experimental outcome was different from both of these predictions. The average fraction of BrdU-positive GCs was highest in the molecular layer when BrdU was injected at P8 (0.37 ± 0.09), which was more than twice as P4 (0.13 ± 0.07) and P12 (0.16 ± 0.12) injections (Fig. 2*C*, top). The mean difference between P4 and P8 injection was significant with the narrow confidence interval relative to the difference, whereas there was no significant difference between P4 and P12 injection (Fig. 2*C*, bottom). These results indicate that mGCs are not merely the last group of GCs that exit the mitotic cycle. Thus, although mGC production may be slightly delayed from regular GCs in the GC layer (rGCs), the timing of mGC production appears to be largely overlapped with rGC production.

### mGCs are stable components of the cerebellar cortex

**Figure 3.**
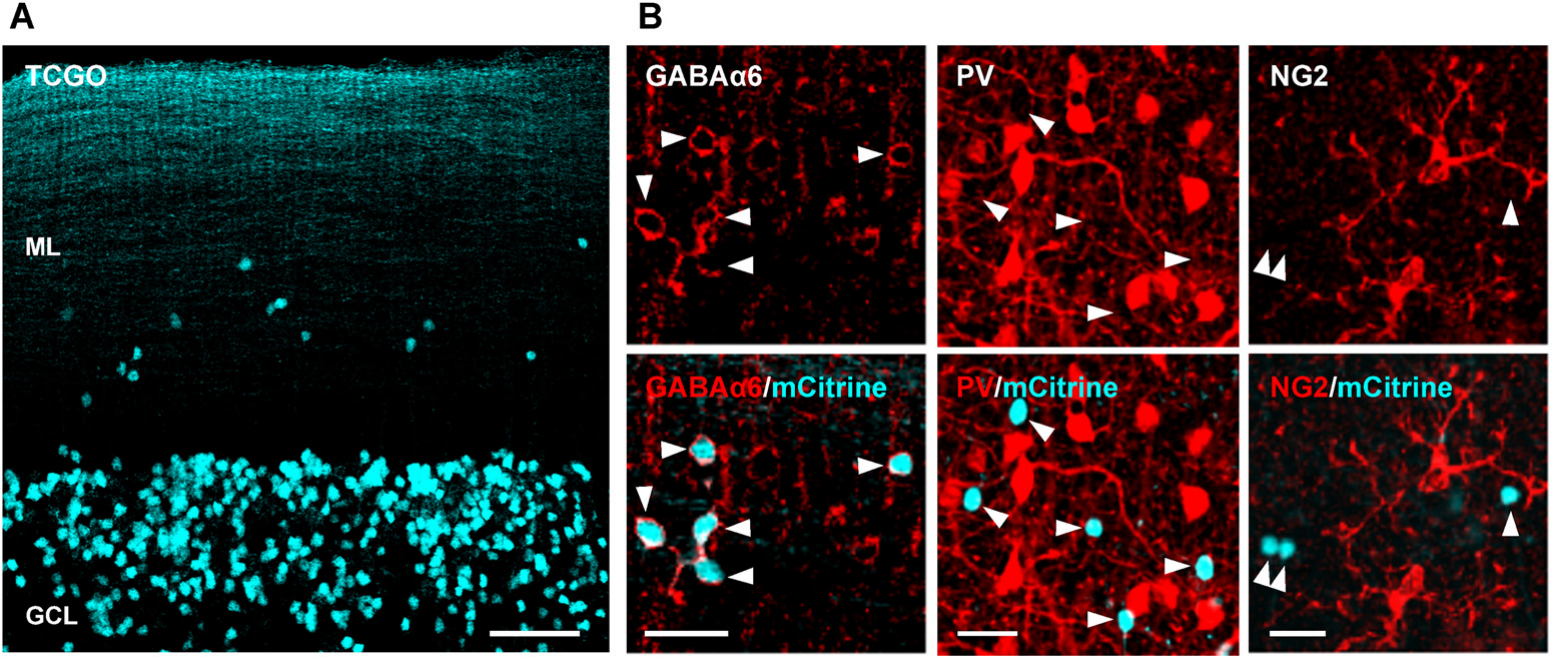
mCitrine exclusively labels mGCs in the molecular layer of TCGO mice. ***A***, The cerebellar cortex of TCGO mice. mCitrine-expressing cells (cyan) are seen in the molecular layer (ML) and GCs layer (GCL). Scale bar: 50 μm. ***B***, The molecular layer of TCGO mice was immunostained with antibodies for GABA_A_Rα6 (left column), PV (middle column), or NG2 (right column). The merged images (bottom row) show the mCitrine signal and the antibody signal in cyan and red, respectively. The arrowheads indicate the location of mCitrine-expressing cells. Note that mCitrine-expressing cells are co-labeled only with the anti-GABA_A_Rα6 antibody. Scale bar: 20 μm.

It is yet to be known whether mGCs are late migrating cells, dying over time because of incorrect placement, or stable components in the molecular layer. To determine which of these possibilities is the case, mGCs must be visualized in living specimens. We, therefore, characterized the transgenic mouse line called TCGO, in which rGCs are labeled by the fluorescent protein mCitrine (Huang et al., 2013; Shima et al., 2016; Dhar et al., 2018). Consistent with the previous report, rGCs were sparsely labeled by mCitrine in TCGO mice (Fig. 3*A*). Besides, we found mCitrine-expressing cells in the molecular layer as well (Fig. 3*A*).

To confirm the identity of mCitrine-expressing cells in the molecular layer, we performed immunohistochemistry using antibodies for GABA_A_Rα6, PV, and NG2, a marker for NG2 glial cells that are known to have their somata in the molecular layer (Levine and Card, 1987; Lin et al., 2005). Only GABA_A_Rα6 immunosignals colocalize with mCitrine-expressing cells, indicating that they are mGCs, not MLIs or NG2 glial cells (Fig. 3*B*). Because of the sparse labeling nature of TCGO mice, not all mGCs or rGCs express mCitrine. Regardless, the TCGO mouse line provides a powerful experimental tool to characterize mGCs in living specimens.

**Figure 4.**
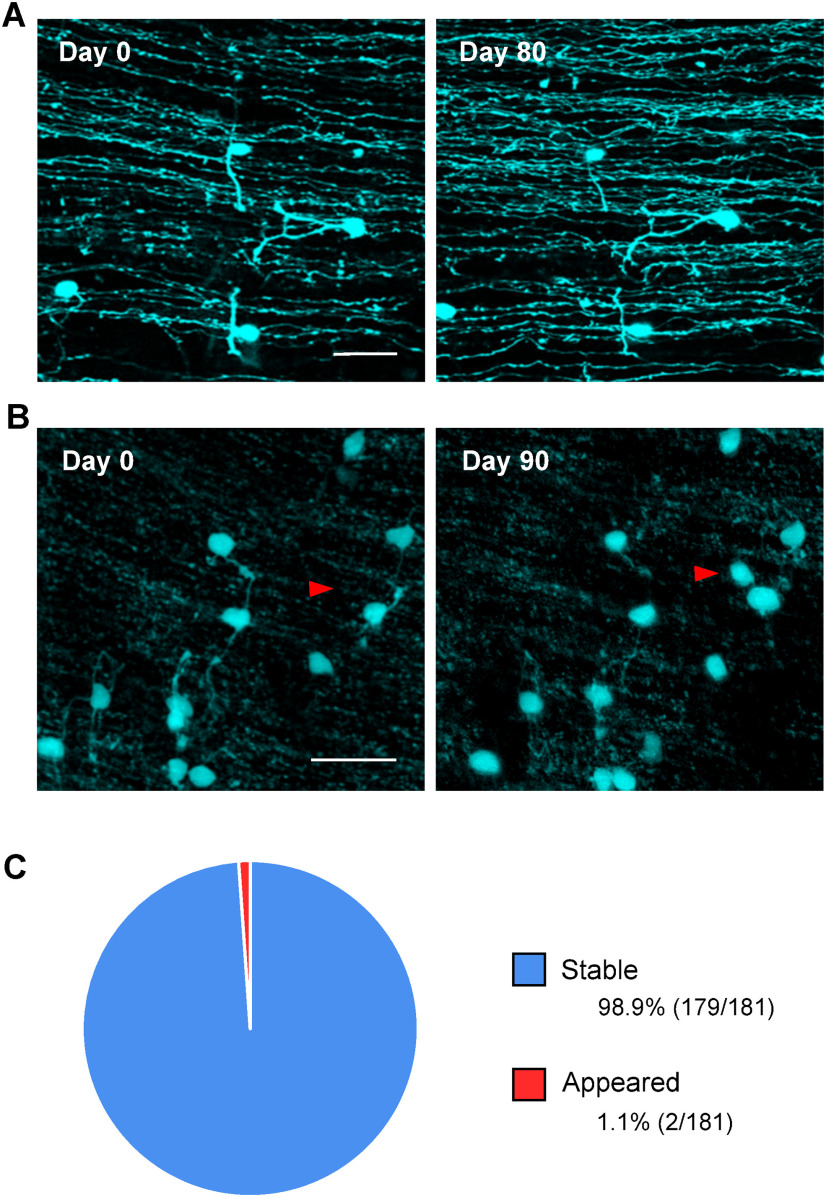
Long-term time-lapse *in vivo* imaging of mGCs. ***A***, ***B***, Representative images of mGCs *in vivo*. Images taken on the first day of imaging (day 0) are compared with the image taken 80 d later (***A***) and 90 d later (***B***). Axons running laterally in the images are parallel fibers. Red arrowheads in ***B*** indicate the mGC that did not exist on day 0 but appeared on day 90. Note that the images are maximum projections; hence, a small angular difference in the optical axis sometimes causes false changes in the projected images. The appearance of the mGC, indicated by the red arrowheads, was confirmed in the z-stack. Scale bar: 20 μm. ***C***, The fractions of stable and newly appeared mGCs were quantified in 181 mGCs (from 10 mice) that were imaged longer than one month. Only two mGCs appeared, and no mGC disappeared. Scale bar: 20 μm.

To determine the stability of mGCs, we performed two-photon *in vivo* time-lapse imaging using adult TCGO mice. Since rGCs send their axons, called parallel fibers, to the molecular layer (Altman and Bayer, 1996; Palay and Chan-Palay, 1974); mGCs can be easily identified by their co-presence with parallel fibers (Fig. 4*A*,*B*). Among 181 mGCs (from 10 mice) imaged over one month or longer, only two mGCs newly appeared (Fig. 4*C*). None of the mGCs imaged at the first time point disappeared later. It remains unclear whether the new mGCs actually appeared or mCitrine-nonexpressing mGCs somehow started to express mCitrine. However, almost all mGCs barely changed their positions or shapes, suggesting that they were stable components of the molecular layer.

### mGCs discharge action potentials

**Figure 5.**
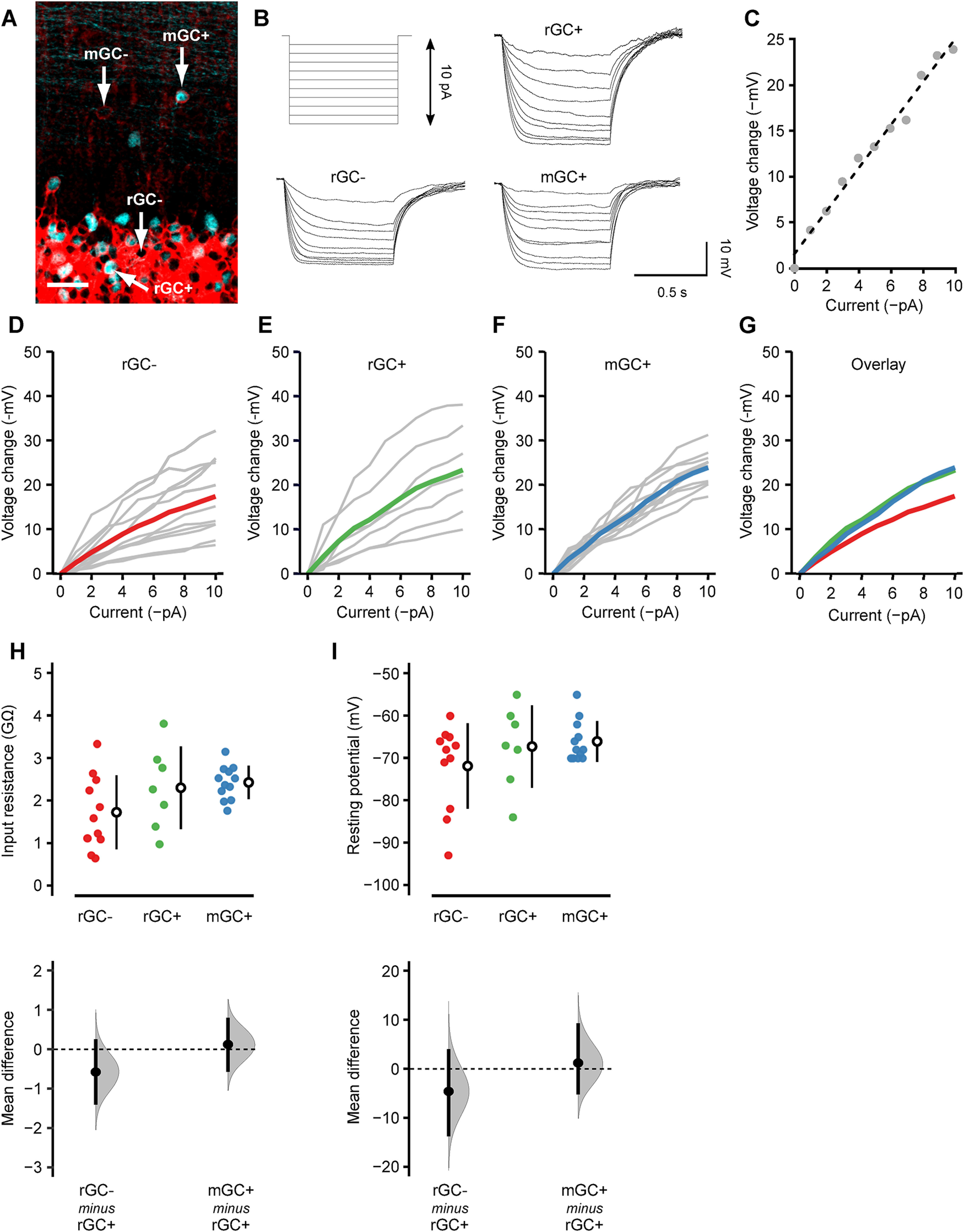
Passive membrane properties of mGCs. ***A***, GABA_A_Rα6 (red) and mCitrine (cyan) double-labeling in a fixed tissue to show the identity of recorded cells. Whole-cell current-clamp recordings were made from rGC– (mCitrine-nonexpressing rGC), rGC+ (mCitrine-expressing rGC), and mGC+ (mCitrine-expressing mGC). mGC– (mCitrine-nonexpressing mGC) is invisible in living tissues, thus not recorded. Scale bar: 20 μm. ***B***, Hyperpolarizing current steps (1-pA increment) injected into the cells (top, left) and the resultant membrane hyperpolarization in a rGC– (bottom, left), rGC+ (top, right), and mGC+ (bottom, right). ***C***, A representative current–voltage (I–V) relationship (gray dots) and the linear regression line (dashed line). The input resistance of each cell was obtained as the slope of this linear regression line. ***D–F***, The I–V relationship of rGC– (***D***), rGC+ (***E***), and mGC+ (***F***). Gray lines represent individual cells, and the colored lines represent the population average. ***G***, The population averages of rGC– (red), rGC+ (green), and mGC+ (blue) are overlayed. ***H***, ***I***, The input resistance (***H***) and resting membrane potential (***I***) of rGC– (red; *n* = 11), rGC+ (green; *n* = 7), and mGC+ (blue; *n* = 12) were shown, and the mean differences between rGC+ versus rGC– and rGC+ versus mGC+ were estimated by bootstrap resampling. In the top panels, the closed circles indicate individual cells. The open circles and vertical lines indicate the mean and SD, respectively. In the bottom panels, the gray curves indicate the resampled distribution of the mean difference between rGC+ versus rGC– (rGC– minus rGC+) and rGC+ versus mGC+ (mGC+ minus rGC+). The closed black circles and vertical lines indicate the observed mean difference and 95% confidence interval, respectively. The horizontal dashed line is the line of zero mean difference.

Because of the uncommon location of mGCs and their morphology, it has been thought that mGCs are erroneous neurons with their maturation stalled midway (Berciano and Lafarga, 1988). However, no functional characterization has yet been made. Using cerebellar slices acutely prepared from TCGO mice, we performed whole-cell patch-clamp recordings from visually identified mGCs. Recordings were made from mCitrine-expressing mGCs (mGC+), mCitrine-expressing rGCs (rGC+), and mCitrine-nonexpressing rGCs (rGC–; Fig. 5*A*). Although mCitrine-nonexpressing mGCs were not identifiable, thus not recorded, the comparison between rGC+ and rGC– was expected to provide insight into potential differences between mCitrine-expressing and nonexpressing GCs, if any.

We held the cells with current-clamp mode and injected a hyperpolarizing current step (0–10 pA) with 1-pA increment (Fig. 5*B*). The voltage-current relationship was obtained in each cell (Fig. 5*C–G*), and the input resistance was measured as the slope of the linear regression line (Fig. 5*C*,*H*). The input resistance showed substantial cell-to-cell variation in rGC– (1.65 ± 0.85 GΩ) and rGC+ (2.30 ± 0.98 GΩ), as indicated by the relatively large SD compared with the mean. On the other hand, the cell-to-cell variation was small in mGC+ (2.42 ± 0.39 GΩ). The difference in variation was statistically significant (*p* = 0.028, Bartlett’s test), suggesting that mGCs are more homogeneous than rGCs.

We also compared the mean input resistance between rGC+ versus rGC– and rGC+ versus mGC+ because rGC+ shares a common property with both cell types (rGC–: location, mGC+: mCitrine expression); thus, suitable as the control group for our statistical analysis. The 95% confidence interval of the mean difference includes zero in both rGC– and mGC+, indicating that the mean input resistance was not significantly different among the cell types (Fig. 5*H*). The resting membrane potential, measured as the voltage without a holding current, was not significantly different among the cell types either (Fig. 5*I*). These results suggest that, although mGCs may be more homogenous than rGCs, their passive membrane properties are as mature as rGCs.

**Figure 6.**
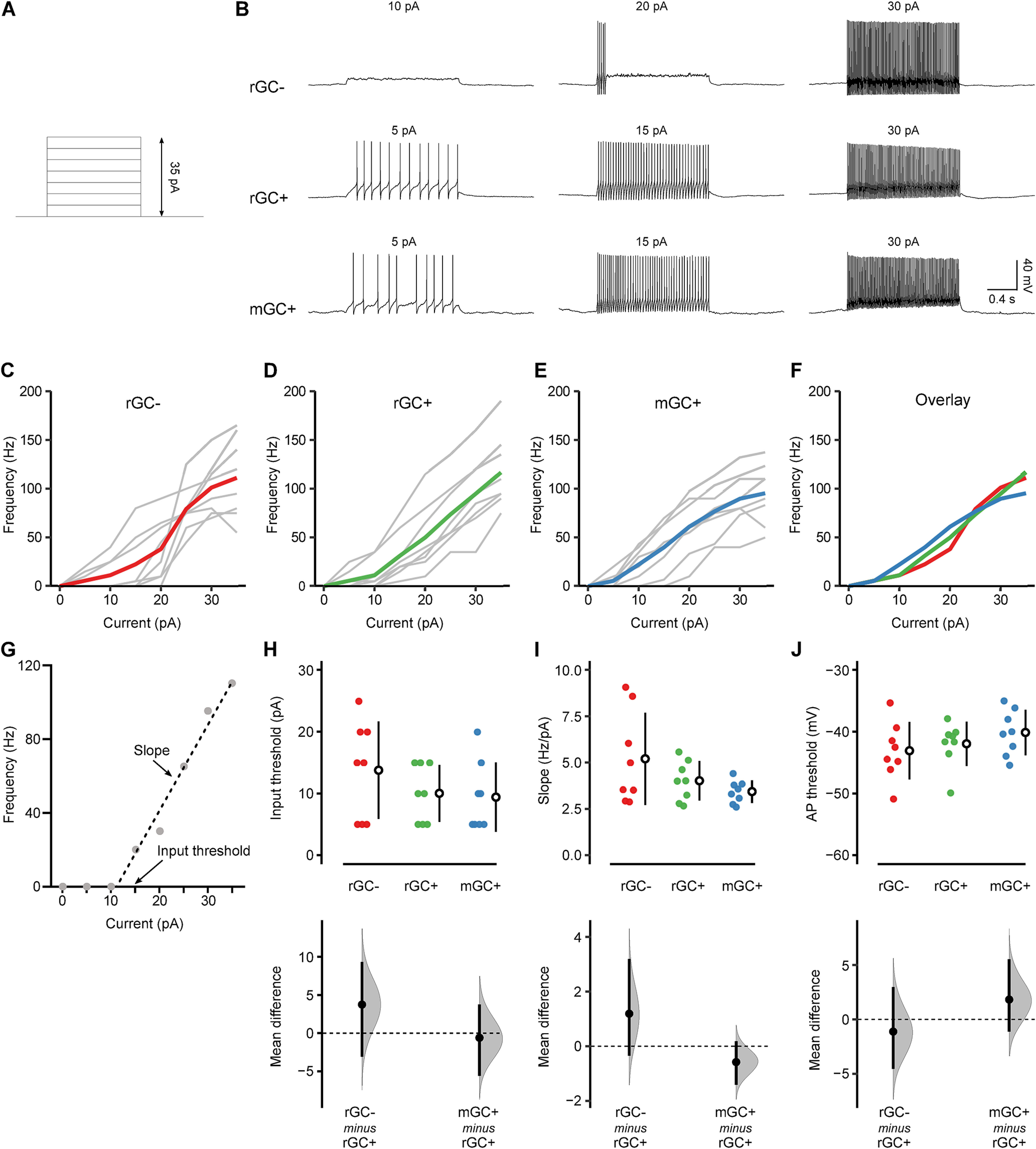
Excitability of mGCs. ***A***, Depolarizing current steps (5-pA increment) injected into the cells. ***B***, The action potential discharge in a rGC– (top), rGC+ (middle), and mGC+ (bottom) on current injection. The amplitude of the injected current is shown above each trace. ***C–E***, The frequency of action potential discharge as a function of injected current in rGC– (***C***), rGC+ (***D***), and mGC+ (***E***). Gray lines represent individual cells, and the colored lines represent the population average. ***F***, The population averages of rGC– (red), rGC+ (green), and mGC+ (blue) are overlayed. ***G***, A representative current-frequency relationship (gray dots) and the linear regression line (dashed line). The linear fit was performed between the maximum current that did not evoke action potential (10 pA in this cell) and the current that elicited the highest frequency of action potentials (35 pA in this cell). The input threshold is the minimum current that evoked action potentials (15 pA in this cell). ***H–J***, The input threshold (***H***), the slope of the linear regression line (***I***), and action potential threshold (***J***) of rGC– (red; *n* = 8), rGC+ (green; *n* = 8), and mGC+ (blue; *n* = 8) were shown, and the mean differences between rGC+ versus rGC– and rGC+ versus mGC+ were estimated by bootstrap resampling. In the top panels, the closed circles indicate individual cells. The open circles and vertical lines indicate the mean and SD, respectively. In the bottom panels, the gray curves indicate the resampled distribution of the mean difference between rGC+ versus rGC– (rGC– minus rGC+) and rGC+ versus mGC+ (mGC+ minus rGC+). The closed black circles and vertical lines indicate the observed mean difference and 95% confidence interval, respectively. The horizontal dashed line is the line of zero mean difference.

We next examined the firing properties of mGCs by injecting a depolarizing current step (0–35 pA) with 5-pA increment (Fig. 6*A*). All three types of GCs discharged action potentials, and the firing frequency increased as the amplitude of the injected current increased (Fig. 6*B–F*). Consistent with previous studies (Brandalise et al., 2016), there was substantial cell-to-cell variation in the minimum current required to elicit action potentials (input threshold) and the slope of the current-frequency relationship (Fig. 6*G*). Similar to the input resistance, mGCs showed the smallest variation in the slope (Fig. 6*I*), and the difference in variation was statistically significant among the cell types (*p* = 0.002, Bartlett’s test). It suggests that the input-output relationship, i.e., how the input strength is converted to the firing frequency, is relatively homogeneous in mGCs. However, there was no significant difference between rGC+ versus rGC– or rGC+ versus mGC+ in the mean input threshold (Fig. 6*H*), slope (Fig. 6*I*), or action potential threshold (Fig. 6*J*) as shown by the 95% confidence interval including zero in all the cases. These results suggest that, although the physiological properties of mGCs may be more homogenous than rGCs, their intrinsic excitability is as mature as rGCs.

## Discussions

This study reevaluated mGCs with modern experimental tools that were not available when previous studies characterized them several decades ago. We used the anti-GABA_A_Rα6 antibody, a GC-specific marker, to quantify the distribution and abundance of mGCs. We also used the TCGO transgenic mouse line to analyze for the first time the functional properties of mGCs. Our data show that the number of mGCs is estimated to be ∼70% of stellate cells or basket cells in the posterior lobe and flocculonodular lobe, and they discharge action potentials in a similar fashion as rGCs. These results suggest that mGCs are one of the major neuronal subtypes in the molecular layer of the posterior cerebellum, potentially forming a previously unconsidered synaptic circuit in the cerebellar cortex.

### Quantification of mGCs and MLIs

An anti-GABA_A_Rα6 antibody has been used in numerous studies since it was established as a GC-specific marker. But, to our knowledge, those studies barely described mGCs in the normal adult cerebellum. It is probably because the intense GABA_A_Rα6 immunoreactivity in the GC layer overshadows the signals in the molecular layer; thus, mGCs have not received much attention. rGCs are densely packed in the GC layer, and they are the most abundant neurons in the mammalian brain. It could be possible to argue that mGCs have little contribution to cerebellar function because the fraction of mGCs is small compared with rGCs. However, GC activity is sparse, and only a subset of rGCs convey action potentials to their postsynaptic targets on sensory stimulation (Chadderton et al., 2004; Jörntell and Ekerot, 2006; Ruigrok et al., 2011; Wilms and Häusser, 2015). Therefore, mGC activity might not be overwhelmed by rGCs, and it is more likely the case if mGCs and rGCs receive different types of excitatory inputs.

When we combine all images taken from the entire cerebellum, the mean density of MLIs and mGCs is 110.6 ± 30.4 × 10^3^/mm^3^ and 29.4 ± 14.8 × 10^3^/mm^3^, respectively. A previous study using Lapham’s staining and hematoxylin-eosin staining estimated that the mean density of MLIs was 83.8 × 10^3^/mm^3^ in mice (Sturrock, 1989), which is reasonably similar to this study. On the other hand, another study using anti-PV antibody estimated that MLI density in mice was ∼90/0.1 mm^2^ (Collin et al., 2005). If we assume that the density is isotropic, the 2D density of 90/0.1 mm^2^ (900/mm^2^ = 30/mm) is converted to the 3D density of 27 × 10^3^/mm^3^. While this value is substantially smaller than this study and Sturrock, 1989; the accuracy of such conversion is unclear. Besides, obtaining consensus cell density data has been very challenging in general (Keller et al., 2018). Therefore, we argue that the mGC/MLI ratio is more reliable than the absolute density of mGCs and MLIs.

The mGC/MLI ratio was significantly different between the anterior and posterior cerebellum. Although its functional implication is unknown, previous studies also reported other types of difference along the anterior-posterior axis of the cerebellar cortex. For example, a distinct isoform of phospholipase C is used in the anterior and posterior cerebellum for the proper maturation of climbing fiber-Purkinje cell synapses (Kano et al., 1998). Unipolar brush cells, excitatory interneurons in the GC layer, are more prevalent in the posterior cerebellum, particularly in the flocculonodular lobe (Harris et al., 1993; Mugnaini et al., 2011). Still, differences along the anterior-posterior axis are lesser-known than those along the mediolateral axis of the cerebellum (Apps and Hawkes, 2009; Apps et al., 2018). The uneven distribution of mGCs, MLIs, and their ratio reported herein is intriguing because it highlights the heterogeneity of the cerebellar cortex along the anterior-posterior axis.

### Developmental profile of mGCs

It was previously speculated that mGCs are the group of GCs that exits the mitotic cycle too late and cannot complete the migration (Berciano and Lafarga, 1988). To test this hypothesis, we performed BrdU cell proliferation assay. Previous studies showed that all GCs are proliferating at P4 (Fujita, 1967), whereas our data showed that ∼50% of rGCs, not 100%, were BrdU-positive when BrdU was injected at P4. This apparent discrepancy is likely because of the frequency of proliferation marker injection. The frequency of injection is critical because the proliferation marker signal gradually decreases and eventually becomes undetectable as the labeled cells undergo more mitotic cycles after injection. We injected BrdU only 1 d in this study; hence even if all GCs were labeled at P4, cells that kept proliferating would eventually be undetectable. On the other hand, previous studies repetitively injected 3H-thymidine over many days until the time of tissue collection at P20 (Fujita, 1967). Therefore, the 3H-thymidine signal was not diluted by proliferation.

To detect GCs that exit the mitotic cycle last, P12 injection should be most efficient because labeled GCs experience fewer mitotic cycles than P4 and P8 injection. However, our data showed that P8 injection was most efficient in labeling mGCs, suggesting that mGCs are not merely the cells that exit the mitotic cycle last. Although it remains unclear why some GCs in the normal cerebellum stop migration in the molecular layer, factors other than timing appear to affect the expression of migratory cue molecules.

rGCs undergo extensive dendritic refinements once they migrate into the GC layer. While immature, postmigration rGCs have >10 dendrites, these nascent dendrites are subsequently pruned such that mature rGCs have an average of four dendrites with claw-like terminals. (Altman, 1972; Dhar et al., 2018). We did not examine whether mGCs also undergo similar dendritic refinements because reconstructing the entire mGC structure is challenging in our specimens because of the parallel fiber labeling (Fig. 4). However, a previous study using Golgi staining showed that mGCs have fewer dendrites than rGCs, and their dendrites often lacked claw-like terminals (Lafarga and Berciano, 1985). These differences might be caused by different presynaptic inputs available in the molecular layer and GC layer.

### Motility of mGCs

During the formation of the cerebral cortex, radial glial cells guide the migration of cortical neurons, and they disappear once the migration completes (Gadisseux et al., 1990). On the other hand, Bergmann glia, which guides GC migration in the cerebellum, persistently presents throughout the animals’ lives (Yuasa et al., 1996; Dahmane and Ruiz-i-Altaba, 1999; Rahimi-Balaei et al., 2018). Therefore, neuronal migration in the cerebellum might occur beyond the developmental period. Indeed, a relatively recent study suggested that neuronal proliferation and migration in the rabbit cerebellum continue until around six months old, much later than the disappearance of the external GC layer around five weeks old (Ponti et al., 2008). Traditionally, GC migration has been thought to be complete by the end of the third postnatal week in rats and mice (Altman and Bayer, 1996; Rahimi-Balaei et al., 2018). However, a small number of late migrating GCs, even if they exist, are difficult to detect by between-animal comparisons of fixed tissues. To examine whether mGCs are on their way to the GC layer or stay in the molecular layer, we performed two-photon imaging and followed the same mGCs for days to months *in vivo*. Our data demonstrated that mGCs are not migrating. Thus, mGCs stay in the molecular layer despite the persistent presence of Bergmann glia.

### Physiologic properties of mGCs and comparison to other ectopic neurons

While previous studies analyzed the electrophysiological properties of immature GCs before or during the migration in the molecular layer, no functional characterization has yet been made for mGCs in the adult cerebellum. We took advantage of GC-specific labeling in TCGO mice and performed the first-ever electrophysiological recordings from mGCs. One of the most significant findings is that the intrinsic membrane properties of mGCs are as mature as rGCs. In particular, the capability of mGCs to discharge action potentials is crucial because it allows them to be a part of synaptic circuits in the cerebellar cortex.

One unexpected finding is that some physiological properties of mGCs were more homogenous than rGCs as shown by less variation within the group. It may suggest that the functional maturation of individual GCs is influenced by cell-to-cell variation in their developmental process occurring in the GC layer, such as the pruning of excess dendrites. Another interesting observation is that mCitrine-nonexpressing rGCs (rGC–) tend to show slightly different membrane properties and excitabilities from mCitrine-expressing rGCs (rGC+) and mGCs (mGC+). Although the difference was not statistically significant, mCitrine expression might affect the physiological properties of GCs to some extent. Alternatively, mCitrine expression in TCGO mice might not be random but specific to a previously unknown GC subgroup.

Ectopic neurons that have been most extensively studied thus far are GCs in the hippocampal dentate gyrus (DG). Regular DG GCs reside in the GC layer and extend their dendrites in the molecular layer, where they receive excitatory inputs from the perforant path. They send their axons, mossy fibers, toward CA3 regions through the hilus. However, a small population of DG GCs resides in the hilus in normal animals, and the population increases substantially after a seizure (Gaarskjaer and Laurberg, 1983; Parent et al., 1997; Scharfman et al., 2007). Depending on the proximity to the GC layer, those hilar ectopic GCs have their dendrite in the molecular layer or not (Scharfman et al., 2007; Pierce et al., 2011).

Hilar ectopic GCs in the normal animals showed similar intrinsic membrane properties as regular DG GCs (Scharfman et al., 2003, 2007), which is consistent with mGCs in this study. Although little is known about the synaptic connectivity of hilar ectopic GCs in the normal DG, seizure-induced hilar ectopic GCs receive excitatory inputs from mossy fibers, suggesting that they receive primary inputs from other GCs (Pierce et al., 2005).

In the cerebellum, rGCs receive excitatory inputs from mossy fibers (the same name but different from the hippocampal mossy fibers) in the GC layer. Since cerebellar mossy fibers rarely innervate into the molecular layer, we argue that the amount of mGCs seems too many if all of them are innervated by mossy fibers. Indeed, mGC clusters near the pia mater make synapses with mossy fibers, whereas individual mGCs dispersed in the molecular layer do not (Lafarga and Berciano, 1985). Perhaps, individual GCs cannot produce sufficiently strong neurotropic cues to attract mossy fibers.

If mGCs are innervated by nearby axon terminals in the molecular layer, two potential sources of inputs are parallel fibers and climbing fibers (Palay and Chan-Palay, 1974; Altman and Bayer, 1996). Parallel fibers are the axons of rGCs. If mGCs receive parallel fiber inputs, they receive inputs from other GCs, which somewhat resemble seizure-induced hilar ectopic GCs in the hippocampal DG.

Climbing fibers release a massive amount of glutamate, evoking EPSCs via spillover in nearby cells even if they are not synaptically connected. All previously examined neurons and glial cells, whose dendrites or processes reside in the molecular layer, receive climbing fiber inputs via synaptic transmission or spillover (Matsui and Jahr, 2003, 2004; Lin et al., 2005; Szapiro and Barbour, 2007; Coddington et al., 2013; Nietz et al., 2017). Therefore, it is hard to imagine that only mGCs are somehow sequestered from the spillover-mediated volume transmission in the molecular layer. Identifying presynaptic inputs of mGCs is the next crucial step for understanding their potential roles in cerebellar synaptic circuitry.

## Synthesis

Reviewing Editor: Lynda Erskine, University of Aberdeen Decisions are customarily a result of the Reviewing Editor and the peer reviewers coming together and discussing their recommendations until a consensus is reached. When revisions are invited, a fact-based synthesis statement explaining their decision and outlining what is needed to prepare a revision will be listed below. The following reviewer(s) agreed to reveal their identity: Mary Morrison.

Your manuscript has been reviewed by two experts in the field. Both are agreed that the work is interesting and novel. However, there are a number of aspects of the study that need to be clarified before the manuscript can be accepted for publication. These mainly involve text changes to clarify aspects, of the methods and the interpretation and emphasis of the results.

Below is a synthesis of the reviews including a list of the points that we are all agreed are essential to address.

General points from reviewer 1:

The paper includes functional characterization of a class of probable granule neurons located in the cerebellar molecular layer (mGCs), not in the granule cell layer (rGCs). mGCs apparently represent about 30% of all cells in the molecular layer. Lafarga and Berciano documented the presence of these neurons by Golgi stain in the 1980s (Lafarga and Berciano, 1985; Berciano and Lafarga 1988), but their time of generation, persistence, and electrical activity were unexplored before this manuscript. The current data hint at a heretofore-unknown possible additional circuit in the cerebellum-but stop short of demonstrating the existence of that circuit.

General points from reviewer 2:

In this study the authors address the question of how ectopic granule cell in the cerebellar molecular layer (mGCs) compare to the regular ones in the granular layer (rGCs). Using the mouse as model organism, they perform extensive morphological and physiological experiments to elucidate the developmental profile and excitability of mGCs. They complement their mostly ex vivo dataset with in vivo longitudinal imaging to track maturation and potential migration. The question is of interest for both the cerebellar and neurodevelopmental field, as well as for all those interested in cell type heterogeneity. There are however a few concerns that needs to be addressed.

Major points (synthesis of comments from both reviewers):

1. The main finding of the paper is that mGCs are not immature, migrating, and/or late developing cells that got randomly left behind. Instead, they appear to be a distinct sub-population of granule cells, similar enough to the regular ones in terms of physiology, but clearly located in a different layer and thus very likely incorporated in different micro-circuits. This is not immediately clear by the title, which should be rewritten to capture the true main finding, and it should be stressed in the discussion.

2. Page 11-12: Proper quantification is important. Triple stain with GAD67, GABAa6 and GFAP or another glial marker, and give exact numbers of what exact percentage of cells in the ML are mGCs. Or if you do not have a 3 laser confocal, do it sequentially. It is very important to show exactly how many mGCs are there compared to MLI and glia, and at the moment the data presented do not quite nail it. If they are the same number of MLI, or even more, then you have characterized a really substantial interneuronal population.

3. While all experiments chasing the developmental question in terms of both birth-dating and physiological maturity are well designed and go in depth to answer the question, the part that deals with synaptic connectivity feels rushed and superficial. The authors clearly know it themselves, and devote a large part of the discussion explaining which other experiments they did to further characterize and substantiate this - experiments that unfortunately did not work, so no data is presented. In my opinion, this suboptimal section is not in line with the overall quality of the paper, and should be removed. I think that there is scope to start a second study to properly chase up the pre- and post-synaptic partners of these mGCs, building on some of the already attempted experiments, and adding, for instance, co-localization analysis on climbing fiber terminals onto mCGs, reconstructions of sparsely labelled mGCs to find their synaptic targets, physiology aimed at dissecting spillover contributions etc. The present paper should properly focus on the main developmental findings, and the discussion could do more to contextualize and generalize them, e.g. in the context of ectopic cells in other brain areas and the cell type/subtype debate.

The vague description in the Discussion of attempts to label climbing fibers and mGCs in the same preparation should be documented in the Methods and Results, or struck from the paper. This is a significant experiment that would be required to claim that the mGCs are actually integrated into a circuit with the climbing fibers.

Likewise for the experiments to label mossy fibers and mGCs in the Discussion-there’s no mention of how many mice, slices, AAV injections, etc. were tried, so it’s not possible to estimate reliability of these statements without including any of these data in the Methods or Results. And this would be a critical experiment to test the claim that mGCs are integrated into cerebellar circuits with mossy fibers.

Page 17 and Figure 7: as mentioned above, I would remove this part altogether. More in detail regarding what is currently presented, I do not think that you cannot say that the failure rate is high but normal in mGCs without patching MLIs (who are known to connect to pfs) in the same prep, stimulating in the same way, measuring the failure rate and comparing statistically. Also, how did you standardize the stimulation protocol? Did you do an increasing range of intensities? Did you move the electrode in multiple locations, and measure the distance from recorded mGC? For Cfs, why not placing the stim electrode just below the closest PCs and try to evoke a response in mGCs? What about testing spillover with specific pharmacology? Did you record spontaneous activity in between stim protocols?

The cutting orientation (coronal versus parasagittal) may have biased the survival/death of certain afferents, or of the dendritic arbors from the mGCs themselves, skewing the electrophysiological data. But these studies lumped both sets of cutting orientations together. This variable may actually explain why Figure 7 shows 58.3% of the recorded mGCs showed no electrical activity at all. Furthermore, parasagittal cutting would sever parallel fibers, which may explain the paper’s lack of finding paired-pulse facilitation that would have been evidence of rGC parallel fiber connections to the mGC (Figure 7). For these reasons, it may be worth teasing out the results from these two planes of section and presenting them separately.

4. The figures lack consistency and are difficult to interpret visually. For instance, scalebars are sometimes missing, what the colours mean is not indicated in the panel itself, annotations to indicate what the figure is about (e.g. double-labelled cells) are scarce, individual data points are not presented together with all bar plots. Moreover, example pictures and traces are not always appropriately chosen to best illustrate the data. Specifically, Fig 1 could use an additional zoomed out panel to show where we are in the cerebellum and more annotation to explain what the reader is looking at: as first figure in the manuscript, it should be much more intuitive and clearly labelled. Conversely, Fig 2 needs more zoomed-in ones to properly show the nuclear BrdU staining and colocalization with the GABA antibody. In fig 4 I see a cell disappear from panel B, and this is not in line with what it is reported in the text. Figs 5-6 do not present example traces for all three groups, making it impossible to visually evaluate if the spiking and subthreshold behaviours are the same.

5. The number of independent tissue sections, number of mice, and number of individual cells recorded is not always thoroughly documented, across the entire paper, frequently making estimates of confidence in the data challenging. These should be included in the Methods or in the Figure legends for every experiment. One example: on page 6, “quantification was done across multiple z sections...”, but from how many different mice? In how many independent staining runs? etc. Each Figure legend should have such information included for easy comparisons.

6. No information is given about negative controls for the immunohistochemistry experiments, in the Methods section or in the Figures. Inclusion of negative control images would be optimal, to boost confidence in the antibody’s signal above noise to boost confidence in mGC identification.

7. Page 11: you do not show any data for distribution patterns across different lobules, but it would be very interesting to include them if you have them, it’s important to show that mGCs are present everywhere.

8. The BrdU injection protocol results differ from a large body of previous P4 injection labeling studies. This manuscript only found 50% of granule neurons to be BrdU+ with 2 injections at P4 (see Figure 2 and page 19), but decades of previous work found 100%. The authors’ explanation for this discrepancy is not adequate as currently worded.

9. The claim that the large number of mGCs is news is overblown, given Lafarga and Berciano’s earlier Golgi work. This claim should be toned down throughout the paper. Instead of calling mGCs “one of the most abundant cell types in the molecular layer,” perhaps language about mGCs being as prevalent as stellate or basket cells would be more appropriate. Instead of Discussion language about mGCs being “barely describe(d),” or “considered negligible,” aimed at boosting the significance of the current paper, the wording should be changed throughout in light of Lafarga and Berciano’s prior work.

This paper also should mention that Lafarga and Berciano found granule cells in the molecular layer to have less extensive dendritic outgrowth than granule cells in the granule cell layer. Closer examination of planes of section in Lafarga and Berciano’s papers may have provided better guidance as to which plane(s) of section would be optimal to preserve mGC structure and afferents in the current work.

10. On page 14 line 12, the manuscript says “not all GABAARα6 positive cells express mCitrine"-why not? Some explanation seems necessary here. Did using this transgenic line bias selection to a subset of mGCs, possibly obscuring the identification of synaptic partners attempted in later figures?

It is important to know if mGCs are also sparsely labelled by citrine, and therefore if you are recording from a subpopulation. This could be easily quantified in data that you already have by counting the percentage of GABAa6+/citrine+ cells vs. GABAa6+/citrine- cells.

Page 20 bottom: mGCs are not necessarily homogeneous, you may be recording from a subgroup that are citrine+. There could be lots of GC subtypes, including among mGCs if they indeed are so many and present in all lobules

12. The methods need more details, especially when it comes to how images were analysed and quantified:

(i) “The field of view was selected randomly from the entire cerebellum” (p. 7) for counting of GABAARα6+ and BrdU+ cells; again, the number of independent animals, fields of view, sections, etc. is missing.

(ii) Figure 4 has no error bars, raising questions of how many sections from how many independent mice/how many independent sections imaged underly each of its bars. The paper only states that a total of 10 mice contributed to Figure 4 experiments.

(iii) How were the transgenic mice acquired, bred and maintained? By backcrossing hets with Bl6 at each generation?

(iv) Confocal settings are missing: AU, laser power, digital gain and offset, pixel size etc., and how they are standardized across samples.

(v) How were double-positive cells defined? By measuring a mean grey value in Image J and setting a threshold, or by eye? Was this validated? Was it done blind to BrdU injection age?

(vi) The immuno, BrdU and image acquisition paragraphs repeat themselves a lot, they could be merged.

(vii) How did you make sure that those 181 mGCs measured longitudinally were indeed always the same? Coordinates in looking for image area? Blood vessels coordinates? ROI mask matching?

(viii) The intracellular solutions used are very minimalistic. Why were they chosen? How do they compare with the published literature?

(ix) How were ephys data analysed? This does not mean in which program, but how exactly did you extract the numbers that you plot? For example, how did you calculate the slope, or the mean input resistance?

(x) Did you measure series resistance and leak? Did you have any inclusion/exclusion criteria to make sure that the cells are all good patches?

Additional points:

a) On page 13 line13, the manuscript says “the experimental outcome was different from neither of these predictions.” I believe “neither” should read “both.”

b) On page 15 line 3, the claim that “suggesting that they were stably integrated into the synaptic circuits in the molecular layer” should be struck. The data in this paper fall short of supporting this claim. In fact, the majority of the mGCs recorded showed no electrical activity at all (Figure 7, 58.3% or 35/60 cells). To make this claim, the authors need to show that stimulation of a specific presynaptic cerebellar fiber triggers activity in a mGC, which they have not yet done (although according to the Discussion, they did try).

c) On page 20 line 5-6, the claim that “the molecular layer is the final destination of mGCs...” is not supported by data in this paper. The two-photon imaging over time did not go very far into adulthood (Figure 4D), and the paper doesn’t document how many mice were included at each age. This statement needs to be toned down to “may be” the final destination, or struck.

d) Why do you include a single rat picture? Is it necessary? If so, I would prefer to see at least two more biological replicates, and have more details in the methods.

e) Pages 15 and Figure 5: I think that you should move panel I at top of the figure, and also analyse membrane capacitance (as well as mentioning Rs). Cm is an important indicator of cell size, especially in something so compact, and it is important to confirm that Cm is similar across groups - if not, the current clamp experiment need to be normalized for it, and plotted as pA/pF on the x-axis.

f) Pages 16 and Figure 6: you have the data, so why not analysing also the action potential properties (AP height, AHP, half width etc) for the first action potential fired by each cell? Also, if Vm is changing, how can you compare excitability across cells? A neuron sitting at -70mV will respond differently than a neuron sitting at -60mV to an injection of 5pA.

g) Page 18 bottom: if you want to make a point about cell number, do the extra experiment and give an exact proportion of how many cells in the ML are mGC. Then, in my opinion, is not so much about cell number, but connectivity, and you have not described how this cells are embedded into the network.
